# Protein and Mitochondria Quality Control Mechanisms and Cardiac Aging

**DOI:** 10.3390/cells9040933

**Published:** 2020-04-10

**Authors:** Rajeshwary Ghosh, Vishaka Vinod, J. David Symons, Sihem Boudina

**Affiliations:** Department of Nutrition and Integrative Physiology, College of Health and Program in Molecular Medicine, University of Utah, Salt Lake City, UT 84112, USA; Rajeshwary.Ghosh@health.utah.edu (R.G.); vishaka.vinod@utah.edu (V.V.); j.david.symons@hsc.utah.edu (J.D.S.)

**Keywords:** aging, chaperone-mediated autophagy, heart, macroautophagy, mitophagy, protein quality control, ubiquitin proteasome system

## Abstract

Cardiovascular disease (CVD) is the number one cause of death in the United States. Advancing age is a primary risk factor for developing CVD. Estimates indicate that 20% of the US population will be ≥65 years old by 2030. Direct expenditures for treating CVD in the older population combined with indirect costs, secondary to lost wages, are predicted to reach $1.1 trillion by 2035. Therefore, there is an eminent need to discover novel therapeutic targets and identify new interventions to delay, lessen the severity, or prevent cardiovascular complications associated with advanced age. Protein and organelle quality control pathways including autophagy/lysosomal and the ubiquitin-proteasome systems, are emerging contributors of age-associated myocardial dysfunction. In general, two findings have sparked this interest. First, strong evidence indicates that cardiac protein degradation pathways are altered in the heart with aging. Second, it is well accepted that damaged and misfolded protein aggregates and dysfunctional mitochondria accumulate in the heart with age. In this review, we will: (i) define the different protein and mitochondria quality control mechanisms in the heart; (ii) provide evidence that each quality control pathway becomes dysfunctional during cardiac aging; and (iii) discuss current advances in targeting these pathways to maintain cardiac function with age.

## 1. Introduction

It is estimated that older individuals (≥65 years of age) will comprise 20% of the United States (US) population by 2030. Advancing age is a risk factor for cardiovascular disease (CVD). Indeed, heart disease is the leading cause of mortality in the older population [1]. Despite intensive research efforts into this area, the American Heart Association (AHA) reports the prevalence of heart failure is still rising in older individuals [2]. As such, identifying new therapeutic targets is critical for the development of novel effective intervention strategies to treat CVD in older individuals.

Protein homeostasis (proteostasis) is achieved when protein synthesis and degradation are balanced in the cell. When protein synthesis exceeds degradation, accumulation of damaged/misfolded proteins (proteotoxicity) occurs, causing organ dysfunction. Post-mitotic cells with limited proliferative capacity such as cardiomyocytes are particularly reliant upon protein degradation pathways to maintain intracellular protein quality control. Protein degradation pathways that have been conserved throughout evolution are responsible for clearing superfluous, damaged, and/or dysregulated proteins in an effort to maintain cellular homeostasis. Similarly, cardiomyocytes maintain mitochondrial quality through a balance between clearance (mitophagy) and generation of new organelles (biogenesis). An attractive and evolving hypothesis is that repression of protein and mitochondria quality control pathways during cardiac aging contributes importantly to aging-associated cardiac dysfunction. Thus, a better understanding of the molecular mechanisms involved in these degradation pathways during cardiac aging is needed. In addition, targeting protein and mitochondria quality control systems might be a viable therapeutic strategy to delay the onset, attenuate the severity, or even prevent aging-associated cardiac dysfunction.

**Figure 1:**
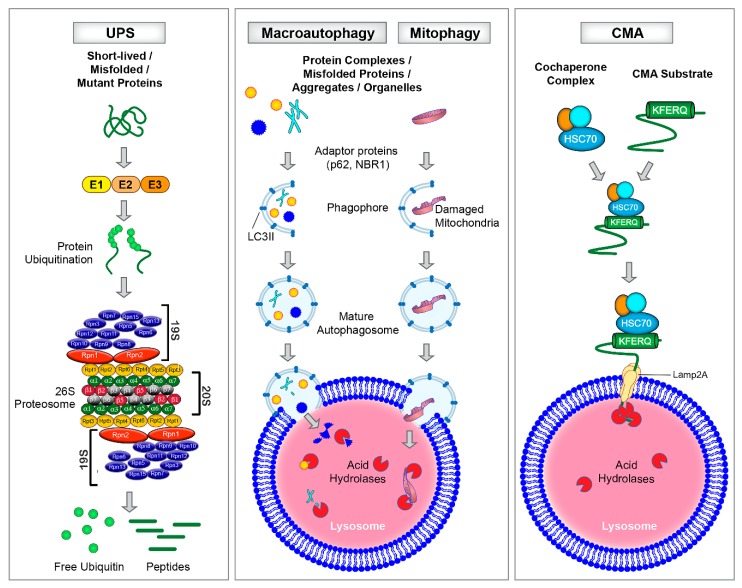
Schematic illustration of different types of protein degradation pathways. *Left*: Ubiquitin Proteasome System (UPS) employs three different enzymes, E1, E2 and E3 to target and tag proteins with four or more ubiquitin molecules. The 26S proteasome is a large protein complex that is comprised of approximately 33 different subunits which form the 20S proteolytic core particle capped by the 19S regulatory lid particle. The 20S core has six catalytic sites that cleave protein substrates. The ubiquitinated proteins are recognized and degraded by the 20S core particle into smaller peptides and the free ubiquitin is available for recycling. *Middle*: Macroautophagy and mitophagy require the formation of double-membrane vesicles called autophagosomes to engulf proteins and damaged mitochondria, for eventual fusion with, and degradation by, the lysosome. *Right*: Chaperone Mediated Autophagy (CMA) functions independently of autophagosomes and employs a co-chaperone complex, Hsc70, and the lysosomal receptor, LAMP2A, for the recognition and degradation of proteins bearing a unique KFERQ-like motif.

**Figure 2:**
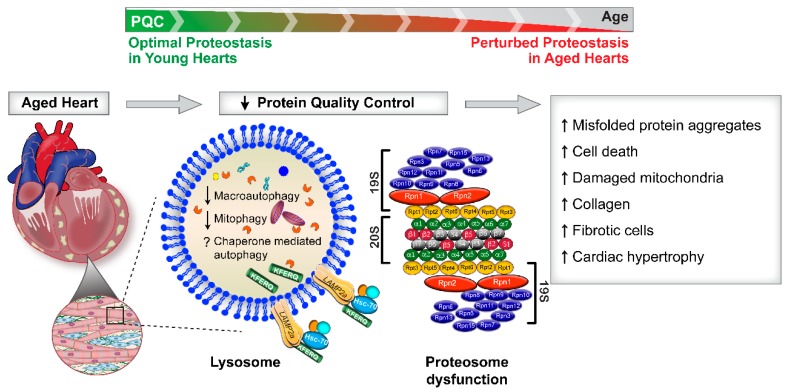
Decline in protein quality control in the aging heart. The heart exhibits a progressive decline in the intracellular protein quality control (PQC) pathways during the aging process. Macroautophagy, mitophagy and chaperone mediated autophagy (CMA) are lysosomal-dependent protein degradation pathways. Strong evidence exists that macroautophagy and mitophagy are decreased in old hearts. At present it is unclear how aging regulates CMA function in the heart. Enzymatic activities of the proteasome are severely reduced in the aging heart. As a consequence of impaired protein quality control, the aging heart accumulates misfolded protein aggregates (proteotoxicity), cell death proteins, and dysfunctional mitochondria. This cardio/proteo-toxic profile is associated with heightened fibrosis, collagen deposition, and cardiac hypertrophy.

**Figure 3:**
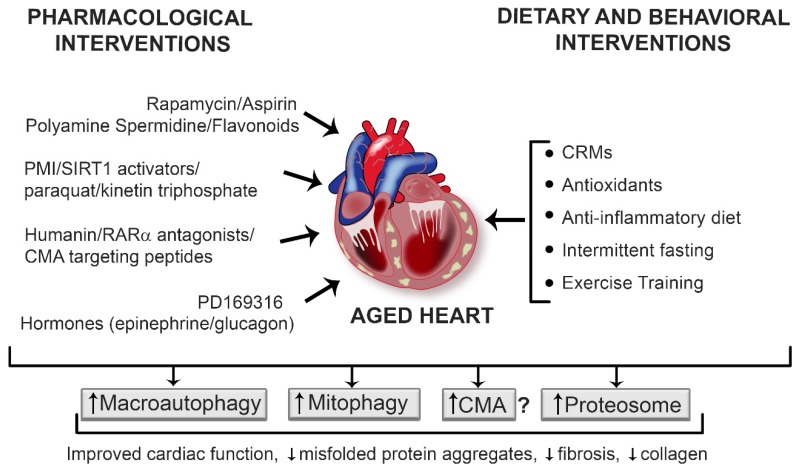
Interventions to upregulate protein quality systems in the aging heart. Nutraceutical (e.g., spermidine), pharmacological (e.g., rapamycin, SIRT1 activators), and dietary and lifestyle (e.g., exercise training, intermittent fasting) approaches have been used to boost protein quality control systems in the heart, in an effort to lessen the severity, delay the onset, or prevent age-associated proteotoxicity and cardiovascular dysfunction. Evidence for the degree of efficacy afforded by each procedure is described in the text. Upregulating the protein quality control mechanisms can prove to be beneficial against various age-related cardiac pathologies. mTOR: mammalian target of rapamycin; PMI: p62-mediated mitophagy inducer; SIRT1: silent mating type information regulation 2 homolog; RARα: retinoic acid receptor alpha; PD169316: p38 MAPK inhibitor; CRMs: caloric restriction mimetics.

Aging is a stress over which we have no control. During this physiological process there is a disruption of cardiovascular homeostasis that can lead to structural and functional changes in the heart, a topic comprehensively reviewed elsewhere [3,4,5,6,7]. Briefly, the aging heart exhibits fibrotic remodeling characterized by an inappropriate accumulation of type 1 collagen in the interstitial compartment of the myocardium [8]. Accrual of type 1 collagen enables ventricular stiffening which can compromise diastolic function. Furthermore, myocardial performance, especially in response to elevated myocardial oxygen demand, is diminished in older individuals, suggesting an additional energetic deficit [8,9]. Concurrent with increased fibrosis and impaired energetics, the aged myocardium displays an elevated pro-oxidant and pro-inflammatory profile [10,11,12], coupled with heightened apoptosis and necrosis [13]. Because misfolded/damaged proteins accumulate during the aging process, a widely held hypothesis is that an imbalance between protein synthesis and protein degradation precipitates age-associated cardiac pathologies. While attenuated matrix metalloproteinase enzyme activity, a key enzyme responsible for degrading collagen, likely is a contributing factor [14], repressed protein degradation pathways also could play an important role. Here we review current understanding of: (1) protein and mitochondria quality control pathways in the heart; (Figure 1) (2) aging-associated disruptions of protein and mitochondria degradation pathways in the heart (Figure 2); and (3) current “therapies” and interventions that target cardiac protein and mitochondrial quality control pathways (Figure 3).

## 2. Protein and Mitochondrial Degradation Pathways in the Heart

The main intracellular protein degradation pathways that have been conserved throughout evolution are the: (i) autophagy-lysosomal pathway, which includes macroautophagy and mitophagy, microautophagy and chaperone-mediated autophagy and (ii) ubiquitin proteasome system (UPS). Key features of these pathways are highlighted in Figure 1.

### 2.1. Autophagy-Lysosomal Pathway

The autophagy-lysosomal pathway can be divided into: (i) macroautophagy and (ii) microautophagy (Figure 1). Macroautophagy (referred to hereafter as autophagy) is a non-selective mechanism of protein degradation which can be activated in the heart by fasting [15] or in cardiomyocytes by nutrient-deprivation [16,17]. Several autophagy-related genes (*Atg*) participate the process of autophagy which involves a series of steps including the initiation, formation, and activation of a double-membrane autophagosome, and vesicle nucleation, elongation, and maturation [18]. Briefly, in mammals, autophagosome formation is initiated by the mammalian Atg1 homolog, UNC-51-like kinase 1 (ULK-1) protein kinase complex which recruits Atg9 and activates other autophagy proteins required for autophagosome formation. The next step is autophagosome nucleation which requires the activation of a class III Phosphatidylinositol 3-kinase(PtdIns3K) complex leading to the formation of Phosphatidylinositol 3-phosphate (PtdIns3P). The latter then recruits effector molecules to the pre-autophagosomal structure that is required for autophagosome formation. Elongation and maturation of autophagosomes are completed by two ubiquitin-like conjugation systems: Atg12-Atg5-Atg16 and LC3II (the mammalian homolog of yeast Atg8). Atg3 (an E2-like homolog) together with Atg7 facilitates the lipidation of cleaved LC3I by adding a phosphatidylethanolamine (PE) group to LC3 to form LC3II. LC3II plays important roles in vesicle elongation and substrate selection, and is often used as a marker for autophagy [18].

The matured autophagosome engulfs cytoplasmic proteins and organelles following which the outer membrane of the autophagosome fuses with the lysosome for the final degradation of the sequestered cargo by lysosomal enzymes [18]. Autophagy adaptor proteins including p62/SQSTM1/sequestosome-1 and NBR1 (neighbor of BRCA1 gene 1) play important roles in tethering damaged and redundant proteins and mitochondria to the autophagosome for lysosomal degradation. Both p62 and NBR1 exhibit similar domain features including the presence of a ubiquitin-associated domain (UBA) that binds mono and polyubiquitinated proteins and a short LC3 interacting region (LIR) [19,20]. The presence of these domains enables p62/NBR1 to link ubiquitinated cargoes to the autophagosomes for degradation. Amino acids are generated as byproducts of lysosomal degradation which are efficiently recycled and consumed by the cells for generating new proteins. Thus, autophagy helps in salvaging energy and contributes to cellular energy balance. Unlike autophagy, in microautophagy, proteins are directly engulfed by the invagination or protrusion of the lysosomal membrane and thus, formation of an autophagosome is not required (Figure 1) [21].

The heart is a metabolically active organ with high demand for ATP that is supplied predominantly by mitochondria. Damaged or dysfunctional mitochondria are constantly eliminated by a selective form of autophagy called mitophagy [22]. While other organelles are targeted by autophagy, we focus on mitophagy in this review because of its known role in cardiac aging. During the process of mitophagy, mitochondria are targeted for removal, tethered to the autophagosome, engulfed, and degraded after autophagosome-lysosome fusion (Figure 1). Mitophagy was first discovered in rat hepatocytes wherein autophagy was induced by nutrient deprivation and glucagon administration [23]. It was shown that depolarized mitochondria were engulfed in the acidic autolysosomes wherein they were digested by lyososomal hydrolases [23]. At present, two main mechanisms of mitophagy have been identified including (i) receptor-mediated mitophagy involving Atg32 as the mitophagy receptor (in yeast); and (ii) phosphatase and tensin homolog induced putative kinase 1 (PINK1) and its E3 ligase Parkinson Protein 2, E3 Ubiquitin Protein Ligase (PARKIN)-dependent mitophagy (in mammals) [24,25,26,27]. Under conditions of homeostasis, PINK1 is constantly degraded by mitochondrial processing peptidase (MPP) and presenilin-associated rhomboid-like protease (PARL). Mitochondrial damage is sensed through membrane depolarization which causes PINK1 to accumulate at the outer mitochondrial membrane. When this occurs, PINK1 and PARKIN ubiquitinate several mitochondrial outer membrane proteins including mitofusin 1 and 2 (MFN1 and MFN2), voltage-dependent anion channel (VDAC), some components of the translocase of the outer membrane (TOM) complex, BCL2 Antagonist/Killer 1 (BAK), Mitochondrial Rho GTPase 1 and 2 (MIRO1 and 2), Mitochondrial fission 1 protein (FIS1), and others [26] via a not fully understood cytosolic cascade and the ubiquitinated mitochondrial proteins are then engulfed by the autophagosome and degraded by the lysosome [28]. Mitochondria can be damaged by stresses including hypoxia [29], insufficient substrates for oxidative phosphorylation, and elevated free radical generation, all of which are features displayed by an aging heart [30,31,32].

### 2.2. Chaperone-Mediated Autophagy (CMA)

CMA is a selective degradation pathway that is unique to mammalian cells. Unlike autophagy or mitophagy, CMA does not require autophagosomes for lysosomal degradation of proteins (Figure 1) [33]. Instead, CMA employs a heat shock cognate (Hsc70) for the recognition and targeting of the cytosolic proteins bearing a unique motif biochemically related to the KFERQ peptide sequence [34]. Once targeted, the chaperone bound proteins are trafficked to the lysosomes where they are recognized by the lysosome-associated membrane protein type 2A (LAMP2A). To assist the internalization and degradation of Hsc70-bound proteins, LAMP2A oligomerizes to form a translocon complex [35,36]. What makes CMA unique is its ability to function independently of autophagy to deliver cytosolic proteins to the lysosomes one at a time. Over the past decade, increasing evidence suggests a relevant role for CMA in various pathophysiological conditions including neurological diseases, diabetes, and cancer [37]. However, limited data are available on the role of CMA in cardiac aging [38].

### 2.3. Ubiquitin Proteasome System

The Ubiquitin Proteasome System (UPS) is a major degradation pathway occurring in the cytosol and nucleus of eukaryotic cells which contributes to the bulk degradation of 80% to 90% of intracellular proteins (Figure 1) [39]. The UPS employs three different enzymes: E1: activating enzyme; E2: conjugating enzyme; and E3: ligating enzyme to: (1) target and tag the identified proteins by the covalent linking of multiple ubiquitin molecules (conjugation); and (2) degrade the proteins by the 26S proteasome complex (degradation) [40,41]. The 26S proteasome complex is comprised of the catalytic 20S core particle and the 19S regulatory lid particle [41]. Under physiological conditions, the UPS functions in an ATP-dependent manner to target the ubiquitinated proteins for degradation in the 20S core particle. In response to pathophysiological challenges, the proteasome acts autonomously to degrade disease-causing proteins in an ATP-independent manner [42]. A key difference between proteasomal and lysosomal-mediated degradation is that short-lived proteins (like the tumor suppressor p53) are degraded by the proteasome, whereas long-lived proteins (like myosin and actin) and large protein complexes (like chaperonin containing TCP-1 complex) [43] which cannot traffic to the narrow proteasomal core, undergo lysosomal degradation [44]. Misfolded proteins including the mutant form of crystallin protein and alpha-synuclein mutant protein are degraded by both systems [45,46,47].

### 2.4. The Interplay Among Protein and Mitochondria Degradation Pathways

Since the above-mentioned degradation pathways are vital for maintaining cellular protein homeostasis, it is not unreasonable to hypothesize that an orchestrated interaction occurs among the players under basal conditions and in response to pathological challenges. Cohen-Kaplan et al. [48]. elegantly demonstrated the mechanism through which proteasome function can be regulated by the autophagy pathway. Amino acid starvation enhances polyubiquitination of 19S regulatory particle subunits Rpn13, Rpn10, Rpn1, and Rpn2 of the proteasome [48]. The ubiquitinated proteasome is translocated to the autophagosomes via its interaction with the ubiquitin-associated domain of the adaptor protein p62/SQSTM1, a process that requires collaboration with the autophagosomal protein LC3. This raises the intriguing possibility that autophagy inhibition heightens proteasomal function due to attenuated proteasomal degradation. Many studies have attempted to delineate the mechanism whereby degradation pathways might balance each other primarily in non-cardiac cells and tissues [49,50,51,52,53]. For example, in the neuroblastoma N2a cell line and primary cortical neuronal cells, impairment of proteasome function initiates autophagic flux which is accompanied by activation of phosphorylated p38α (p-p38α)-dependent apoptosis [53]. Interestingly, the mammalian target of rapamycin (mTOR) inhibitor/autophagy activator rapamycin antagonized p38α activation and facilitated cell survival against proteasome impairment. Consistent with these findings, mTOR complex 1 (mTORC1) activation increased proteasome content in mouse embryonic fibroblasts (MEFs) and human HEK293 and HeLa cells in a manner that could be reversed by either rapamycin or mTOR kinase inhibition using PP242 [54]. Thus a reciprocal relationship appears to exist between autophagy and proteasomal degradation wherein each pathway compensates for the loss of the other in a concerted effort to maintain protein homeostasis. Underscoring the evolution of knowledge in this area, evidence exists that autophagy inhibition does not impact proteasomal activity. Instead, p62 accumulation upon autophagy compromise was shown to interfere with the efficient degradation of ubiquitinated substrates to thereby impair UPS performance in HeLa cells and MEFs [55]. Further studies demonstrated the important role for the proteasome in the maintenance of mitochondrial protein turnover and mitophagy. The components of depolarized mitochondria were shown to be differentially degraded by PARKIN either via mitophagy or through the proteasome pathway. Further, proteins in the outer mitochondrial membrane (OMM) and the intermembrane space (e.g. outer membrane protein (Omp25), Tom20, Tom40, Tom70,) of depolarized mitochondria can be degraded by the proteasome, whereas those in the inner mitochondrial membrane (IMM) and the mitochondrial matrix (e.g., The translocase of the inner membrane (Tim)23, Tim17, Complex-III core 1, and Tim44) are degraded mainly by mitophagy in cultured fibroblasts [56]. Interestingly, it was shown that both the 19S regulatory particle and the 20S core subunits of the proteasome localized in part to mitochondria in COS-7 (African green monkey kidney), HeLa or NIH 3T3 (mouse fibroblast) cell lines [57], suggesting a direct interaction between mitochondria and the proteasome.

In the context of CMA function, it was shown that nutritional stress activates both autophagy and the CMA pathway indicated by increased levels of both lysosomal-Hsc70 and LAMP2a [36,58,59]. However, autophagy is activated within 4–6 h of food restriction which then gradually decreases. This reduction in autophagy coincides with an exponential rise in CMA which reached a peak 24 hour post-starvation [60]. Furthermore, siRNA-mediated depletion of LAMP2a to repress CMA reportedly upregulates macroautophagy in mouse fibroblasts [60]. Consistently, knockout of *Atg5* in MEFs increased CMA function, which further supports a direct interaction between the two forms of autophagy [61]. A possible mechanism underlying this crosstalk is the degradation of ULK1 by CMA [62]. As mentioned earlier, the ULK1 complex plays a central role in the initiation stages of autophagy. ULK1 contains two KFERQ-like motifs and immunoprecipitation assays reveal that the CMA components Hsc70 and LAMP2a strongly bind with ULK1 to facilitate its degradation via CMA [62]. These studies demonstrate the significance of a tight coordination between autophagy and CMA in maintaining cellular protein and energy homeostasis. The interplay between CMA and proteasome function has not yet been tested. However, our preliminary data suggest that overexpression of LAMP2a to upregulate CMA significantly decreases proteasomal peptidase activities in primary cardiomyocytes (unpublished data). It is clinically relevant to determine the interplay among the various protein degradation systems in aged myocardium in order to modulate these pathways in a manner that might alleviate aging-related cardiac pathologies. Our ongoing studies are addressing this. 

## 3. Mechanisms Whereby Suppression of Protein Quality Control Pathways Occurs during Cardiac Aging

### 3.1. Autophagy Suppression in Cardiac Aging

Protein quality control is known to decline with aging in the heart (Figure 2). Reduced autophagy in the aging heart has been reported in flies and 20–26 month-old C57BL/6 mice [63,64,65,66,67]. In contrast, other studies using different ages or strains of mice reported unchanged or even enhanced indices of autophagy [68,69]. However, in most of these studies, static autophagy was measured rather than assessing autophagic flux in the presence or absence of lysosomal inhibitors. Despite these discrepancies, genetic approaches to specifically impair autophagy in the heart provide direct evidence for the involvement of this pathway in cardiac aging. In this regard, cardiomyocyte-specific deletion of *Atg5* in mice accelerated cardiac aging as evidenced by reduced contractile function, development of cardiomyocyte hypertrophy, and deposition of fibrosis [63]. Substantiating these findings, additional reports using genetic manipulations to increase mTORC1 activity documented accelerated cardiac aging in mice [70,71,72]. However, the latter results should be interpreted cautiously as mTORC1 might have off-target effects in addition to influencing autophagy. For example, heightened mTORC1 activity can promote protein synthesis which might explain the cardiac hypertrophy phenotype observed in these mice. Nevertheless, investigations employing mTORC1 activation have provided valuable information concerning the importance of this protein in the suppression of cardiac autophagy.

While mTORC1 activation has been implicated in autophagy suppression in the heart with advanced age [64], knowledge concerning novel signaling pathways that are upstream to this autophagy regulator have recently emerged. The evolutionary conserved transforming growth factor beta (TGFB) signaling pathway is involved in many cellular processes including differentiation, apoptosis, and cellular homeostasis [73]. TGFB is activated in the aging heart and has been shown to contribute to increased cardiac fibrosis [8]. Indeed, suppression of TGFB signaling improves cardiac function in aged mice. [74] Chang et al. [75], recently delineated the role of TGFB-INHB/activin signaling in the regulation of autophagy and age-related cardiac dysfunction in *Drosophila melanogaster (D. melanogaster)*. Specifically, cardiac specific knockdown of TGFB-INHB/activin-like protein daw (Dawdle, an activin ligand) activated *D. melanogaster* mTOR complex 2 (mtorc2) signaling, promoted autophagic flux, and preserved cardiac contractility and cardiac output in old flies [75]. In addition to TGFB, inflammation has been directly involved in the suppression of cardiac autophagy with age. The Nucleotide-Binding Oligomerization Domain, Leucine Rich Repeat and Pyrin Domain Containing 3 (NLRP3) inflammasome initiates an inflammatory form of cell death that has been implicated in cardiac disease [76,77,78]. *NLRP3*-deficient (*NLRP3^−/−^*) mice display longer lifespans, preserved cardiac function, and reduced fibrosis when compared to age-matched littermate controls. Importantly, older mice with *NLRP3* deletion had higher Atg12, Beclin-1, and LC3II protein content and reduced p62 levels [79]. Enhanced autophagy in *NLRP3^−/−^* mice was secondary to mTORC1 inhibition [79]. Benefits observed secondary to *NLRP3* suppression make this a promising therapeutic target to attenuate the adverse effects of cardiac aging and extend lifespan. More recently, Rho-associated coiled-coil–containing protein kinases (ROCKs) which are known to play a role in the progression of cardiomyocyte apoptosis under pathological conditions such as pressure overload [80,81] have also been linked to autophagy and cardiac aging. Shi et al. [82], showed that double deletion of *ROCK1* and *ROCK2* isoforms in cardiomyocytes protected mice from age-associated cardiac dysfunction. Specifically, compared to age-matched wildtype controls, 18 month-old mice with cardiomyocyte deletion of *ROCK1/2* had reduced collagen deposition and cardiac fibrosis. Cardiac *ROCK1/2* double knockout (DKO) mice had higher LC3II protein expression in response to 24 hours food restriction. The increase in autophagy observed in DKO mice was associated with increased phosphorylation of AMP-activated protein kinase (AMPK) and decreased phosphorylation of mTORC1 [82]. Of note, the authors demonstrated that single deletion of *ROCK2* impaired autophagy secondary to increased AKT/mTORC1/ULK1 signaling, and this was associated with cardiac fibrosis. In contrast, *ROCK1* deletion was shown to be protective in the context of doxorubicin cardiotoxicity, partly due to Beclin 1-mediated autophagy initiation. Collectively, this study reveals opposing roles of different ROCK isoforms in autophagy regulation.

Under homeostatic conditions a balance exists between pro-oxidant generation and anti-oxidant defense mechanisms in all tissues. ROS trigger autophagic activity in order to clear oxidized and misfolded proteins [83]. When redox imbalances occur, ROS have the potential to repress autophagy [84]. Since perturbations in cellular redox state strongly associate with aging-related cardiac pathologies [85], it is possible that suppression of autophagy in the aged heart might be secondary to the oxidation of autophagy-related proteins. Proof of concept exists from work on the vasculature, where oxidation of autophagy proteins has been reported. Specifically, mouse aorta exhibit hyperoxidation, and protein carbonylation in response to starvation [86]. Furthermore, HEK cells exposed to H_2_O_2_ exhibited oxidation of the catalytic cysteine thiols on Atg7 and Atg3 [86], resulting in repressed LC3 lipidation and compromised autophagosome maturation. These data provide proof of concept that age-associated ROS production and subsequent oxidation of autophagy-related proteins might impair autophagosome formation and the accumulation of damaged proteins leading to cardiac dysfunction during aging.

Aged hearts often exhibit proteotoxicity characterized by an accumulation of misfolded, mutant, and ubiquitinated protein aggregates (Figure 2) [87,88]. Proteomic profiling performed on cardiac aggregates from old mice has revealed accumulation of many proteins that are components of the ubiquitin-proteasome system and autophagy degradation pathway [88]. Mutations in *LMNA*, which encodes nuclear *Lamin A/C*, precipitates a premature aging syndrome called Hutchinson-Gilford Progeria (HGPS) that is characterized by the accumulation of protein aggregates [89]. Of particular relevance to this review, a common manifestation of *Lamin A/C* mutations and the subsequent accumulation of progerin caused cardiac dysfunction manifested by atrial arrhythmia and hypertrophic cardiomyopathy [90,91]. Evidence supporting this link is provided by a fly model of cardiolaminopathy exhibiting repressed autophagy, accumulation of cytoplasmic *Lamin C* aggregates, impaired cardiac function, severe myofibrillar degeneration, nuclear morphological defects, and evidence of oxidative stress [92]. Cardiac dysfunction was associated with nuclear enrichment of Cap and collar C (CncC) [the orthologue of mammalian nuclear erythroid 2-related factor 2 (Nrf2)], suggesting cellular redox imbalance in flies with *Lamin C* mutations. Upregulating autophagy by *Atg1* overexpression in this model attenuated cytoplasmic *Lamin C* accumulation, improved nuclear morphology, promoted myofibrillar organization, and ameliorated cardiac dysfunction [92]. Overall, this study demonstrated that upregulating autophagy improved the clearance capacity of cardiac cells to an extent that restored cardiac function.

### 3.2. Mitophagy and Cardiac Aging

Maintaining mitochondrial quality and quantity is integral to generating sufficient ATP to sustain myocardial function both at rest and in response to elevated myocardial oxygen demand. Cardiomyocytes regularly recycle/degrade damaged and dysfunctional mitochondria by a specialized form of autophagy termed mitophagy. Dysregulated mitophagy has been reported in cardiac pathologies [93,94,95,96], including cardiac hypertrophy in mice [63,97] and dilated cardiomyopathy in flies [98,99]. *PINK1* mutations are a dominant feature of age-related diseases including Parkinson’s and PARKIN deficiency is associated with distorted mitochondrial morphology, enhanced mitochondrial DNA mutation rate, and premature cardiac aging [67]. The same study also showed that cardiac aging could be reduced by increasing *PARKIN* expression, suggesting that restoring mitophagy may be a therapeutic avenue to slow aging in the heart. While PINK and PARKIN have been predominantly targeted to manipulate mitophagy, upstream regulators of this process also might be of interest. For example, overexpressing heat shock protein 27 (HSP27) in old mice restored cardiac PINK and PARKIN protein expression, improved myocardial function, reduced ROS generation, decreased poly-ubiquitinated proteins, and increased Atg13, Vps34, and Rab7 proteins [100]. The Akt family of serine-threonine kinases together with AMPK have been shown to regulate lifespan [64,101]. For example, their role in mitophagy in the heart was recently suggested by the observation that *Akt2* and *AMPK* double knockout mice exhibit distorted mitochondria morphology that was associated with loss of mitophagy regulators PINK1/PARKIN, BCL2 Interacting Protein 3 (Bnip3) and FUN14 Domain Containing 1 (FundC1, a mitochondrial outer membrane protein) [102]. All of these adverse effects were associated with decreased autophagy markers, thus predisposing the heart to age-induced cardiac dysfunction [102]. Xu et al. [103], explored the role of specific autophagy-related proteins (Atg) in maintaining cardiac health and mitochondrial integrity in flies. A targeted screen of 12 Atg RNAi lines crossed with Myocyte enhancer factor 2 under the control of Gal4 driver which is a transcription activator (Mef2-GAL4)> upstream activating sequence (UAS)-Mito-Timer flies was performed. UAS regulatory sequences drive expression of a mitochondria-targeted green fluorescent protein upon synthesis, which converts to red fluorescence upon oxidation over time. This elegant procedure enables a time-dependent tracking of protein expression to occur [104,105]. Flies with knockdown of *Atg2*, *Atg9* and *Atg18* displayed a shorter lifespan confirming the importance of autophagy in aging [103]. *Atg2*, *Atg9*, and *Atg18* RNAi flies exhibited impaired mitophagy as indicated by a reduced number of Mito-Timer pure red puncta in their heart tube [103], distorted mitochondrial morphology, and a decrease in the number of autophagosomes containing mitochondria at 40 days of age. These effects caused by knockdown of *Atg2-Atg18/Atg9* were accompanied by cardiac hypertrophy and a distortion of the heart tube lumen surface. Collectively, important proof of concept is provided from old flies that boosting Atg2-Atg18/Atg9 activity can stimulate mitophagy to an extent that maintains cardiac health.

Evidence is emerging that mitochondrial ROS generation associated with impaired mitophagy might contribute importantly to premature aging and age-associated cardiovascular disorders [106,107]. Identifying the source of mitochondrial ROS may uncover new therapeutic targets to improve aging-related cardiac dysfunction. In this context, the role of a mitochondrial FAD-dependent enzyme, monoamine oxidase-A (MAO-A), was studied in cardiomyocytes [108]. MAO-A is responsible for the generation of ROS in the form of hydrogen peroxide (H_2_O_2_). MAO-A-induced ROS generation is a major driver of cardiac oxidative stress. Upon MAO-A activation, cardiomyocytes exhibit a senescent phenotype evidenced by the upregulation of p21, p15 and p16 expression, increased expression of senescence-associated β-galactosidase (SA-β-gal) activity, and enlarged cells [108] Furthermore, MAO-A activity induced mitochondrial dysfunction indicated by decreased oxygen consumption rate, and impaired PARKIN-mediated mitophagy. Conversely, restoration of mitophagy by overexpressing PARKIN significantly improved mitochondrial dynamics and decreased the senescent-associated p21 expression and SA-β-gal activity [108] However, because this study did not assess cardiac function concurrently, it is unknown whether activating PARKIN or inhibiting MAO-A activity provides therapeutic benefit with regard to improving cardiac function with advanced age.

On balance, the available literature indicates that the process of mitophagy is impaired in the aging heart [84,109]. Dysfunctional mitophagy, resulting in the accumulation of damaged mitochondria and heightened oxidative stress, may have profound adverse effects on the ability of cardiomyocytes to resist aging. Fortunately, genes and protein targets are being identified that might have therapeutic benefit to increase mitophagy and alleviate cardiac pathologies among older individuals.

### 3.3. Chaperone-Mediated Autophagy (CMA) and Cardiac Aging

Compared to autophagy and mitophagy, the role of CMA in aging-associated cardiac pathologies is less well characterized. Decreased LAMP-2A protein was reported in lysosomes of senescent human fibroblasts [110]. The implication of CMA in aging in general stems from studies involving global LAMP-2 deficient mice, which exhibit premature mortality [111]. LAMP-2 global KO mice develop cardiac hypertrophy and contractile dysfunction by 19 months. A stalled autophagic flux caused by a failure in autophagosome-to-lysosome fusion was observed in several tissues including the heart [111]. While the later study suggests a role of CMA in cardiac aging, LAMP-2 global KO mice had systemic defects that may have contributed to this phenotype. In spite of these data, additional investigations are required to dissect the role of CMA specifically in the aging heart, which may unmask a new therapeutic target for treating age-related cardiac diseases.

### 3.4. Ubiquitin Proteasome System (UPS) and Cardiac Aging

The important role of the UPS in clearing short-lived oxidized, ubiquitinated or misfolded proteins in the context of cardiovascular disease has been demonstrated [112]. A common method for monitoring proteasome activity is the use of fluorescent-tagged substrates specific for the three catalytic sites of the 20S core particle: β5 Chymotrypsin-like, β2 Trypsin-like, and β1 post-glutamate peptide hydrolase (PGPH) or caspase-like [113]. In addition, a transgenic mouse expressing a UPS-specific reporter substrate can be used to measure proteasome activity. The UPS-specific substrate was constructed by modifying an enhanced green fluorescence protein (GFP) via carboxyl fusion of degron CL1, (GFPdgn), to reflect the status of proteasomal degradation [114]. Alterations in GFPdgn protein levels are inversely related to proteasome function. Thus, GFPdgn reporter mice could be a valuable tool to study proteasome function in age-related cardiac pathology. Despite recent advances into the role of the proteasome in cardiovascular diseases, little is known about its function in the context of cardiac aging. Decreased 20S core particle catalytic activities e.g., chymotrypsin-like, peptidylglutamyl-peptide hydrolase, and trypsin-like activities, concurrent with reduced 20S proteasomal content, was observed in hearts from 26 vs. 8 month-old rats, implicating the important role of the proteasome in the heart [115]. Consistent with these findings, hearts from aged mice displayed accrual of oxidized and ubiquitinated proteins [115].

The FOXO family of forkhead transcription factors are key regulators of UPS function through their direct modulation of the expression of genes encoding UPS components and have been linked to organismal longevity [116,117]. FOXOs promote the expression of UPS-related genes including the E3 ubiquitin ligases *atrogin-1 and Murf-1* and modulate the composition of the proteasome [118]. Although FOXOs can modulate aging through other mechanisms, this review will focus on their effect on the UPS in the context of cardiac aging. A study by Blice-Baum et al. [116] showed that modest overexpression of *dFOXO* in *D. melanogaster* counteracted cardiac aging. Most importantly, *dFOXO* transgenic mice exhibited increased expression of genes encoding UPS components. Similarly, RNAi knockdown of UPS genes compromised cardiac function in young flies [116]. Interestingly, when *dFOXO* was excessively overexpressed in flies, heart function was compromised, which may be consistent with other studies suggesting that too much autophagy (which should mimic FOXO overexpression) resulted in heart failure in mice [119]. Another study demonstrated that FOXO4 was both necessary and sufficient to induce expression of the proteasomal component PSMD11 [117]. Further support concerning the importance of the UPS in maintaining cardiac function in response to cardiac stress is provided by loss of function approaches. In this regard, cardiac-specific ablation of component nodes involved in the UPS including *CG9014* (E3 ligase) and *CG3473* (E2 conjugating enzyme) impaired myocardial function and reduced survival in flies challenged with heat stress [116]. Furthermore, microarray data revealed a downregulation of *CG9014* and *CG3473* in aged flies [116]. These data indicate the importance of the UPS in maintaining cardiac homeostasis in the hearts of aged flies. Studies investigating this issue in myocardium from aged mice are warranted.

## 4. Strategies to Enhance Protein Quality Control Pathways

Based on available data, protein quality control pathways are suppressed in the aged heart and treatment strategies designed to restore proteostasis are a novel and reasonable approach to attenuate, delay, or even prevent cardiac aging. Current interventions targeting protein quality control pathways that are pertinent to aging in general and to cardiac aging in particular are summarized in Figure 3.

### 4.1. Pharmacological and Nutraceutical Interventions

Most of the work in this area has focused on inhibiting mTORC1 using rapamycin. For example, mTORC1 inhibition for 3 months using rapamycin reduced age-related cardiac hypertrophy and myocardial dysfunction in mice [120]. Rapamycin, given daily for 8 weeks, also reversed age-associated deterioration in cardiac structure and function in rats [121]. Further, rapamycin treatment for 10-weeks ameliorated age-associated cardiac hypertrophy and diastolic dysfunction [122]. Importantly, these benefits of mTORC1 repression persisted for 8-weeks following the last rapamycin treatment [123]. While rapamycin has been shown to activate indexes of autophagy within 2-weeks of administration, cardiac benefits in the context of aging might also be realized by the ability of mTORC1 inhibition to induce mitochondrial biogenesis at later time points [124]. As such, mTORC1 inhibition via rapamycin might improve both autophagy and mitophagy to an extent that clears damaged mitochondria and rejuvenates healthy ones. The notion of intermittent, short-term treatment with rapamycin also has been explored. This is important because chronic rapamycin administration negatively regulates mTORC2 [125,126]. Additional off-target side effects of longer-term mTOR (mTORC1 and 2) inhibition using rapamycin include decreased insulin sensitivity, glucose intolerance, early onset diabetes, and hyperlipidemia, which have been linked to the diminished mTORC2-mediated Akt phosphorylation [127,128]. Collectively, negative off-target effects of chronic mTOR inhibition using rapamycin likely outweigh the cardiovascular benefits.

The AMP-activated protein kinase (AMPK) is another key regulator of autophagy in mammalian cells and its activity declines in an age-dependent manner [129,130]. Metformin is a first-line medication for treating type 2 diabetes and is a known AMPK activator [131]. Because treatment of diabetic OVE26 mice with metformin activated AMPK and increased autophagy [132], interest sparked with regard to using this compound in the context of cardiac aging. As thought, metformin treatment enhanced contractile function and reduced cardiac fibrosis induced by isoproterenol [133], and protected mice from age-induced ischemic necroptosis [134]. Providing translational relevance, metformin reduced the incidence of age-related cardiovascular disease in type 2 diabetic men [135]. While it is unfortunate that autophagy endpoints were not assessed in that study, it is tempting to speculate that an increase in cardiac autophagy would have been observed. Nevertheless, because metformin use is commonplace in the clinic, and it is associated with metabolic benefits, there is no doubt that determining its efficacy in the context of cardiac aging will be assessed soon. Non-steroidal anti-inflammatory drugs (e.g., aspirin) activate AMPK [136] and exert cardiovascular benefits. Could aspirin-induced cardiovascular benefits be secondary to autophagy stimulation? It is most certain that current studies are investigating this issue and answers are on the horizon.

Natural compounds known to induce autophagy in the heart are sirtuin 1 (SIRT1) activators including resveratrol, SRT1720, the natural polyamine spermidine, flavonoid 4,4′-dimethoxychalcone (DMC), and trehalose. SIRT1 activation by SRT1720 improved cardiomyocyte contractile dysfunction and mitophagy in aged mice [65]. Resveratrol increased the antioxidant reserve capacity and attenuated lipid peroxidation in aged rat hearts [137]. The lifespan extension and cardioprotection exerted by the polyamine spermidine are conserved across species (*D. melanogaster*, mice, and humans). In the fly model, spermidine supplementation lessened age-related cognitive decline and motor impairment, and protected from proteinopathies [138,139,140]. Pertinent to cardiac aging, late-in-life spermidine supplementation in C57BL6 mice reversed age-associated cardiac hypertrophy and improved diastolic and contractile function [141]. Furthermore, spermidine intake improved cardiomyocyte function and reduced cardiac inflammation in old mice. Interestingly, all spermidine-related benefits were nullified in cardiomyocyte-specific Atg5 KO mice, suggesting a requisite role for intact cardiac autophagy in this context. Of translational importance, a recent epidemiological study reported that spermidine intake associated positively with increased survival in humans [142]. Notably, a clinical trial is ongoing to test the efficacy of spermidine on cognitive function and mortality in humans [143]. DMC has life extending properties in yeast, flies, and mice, together with anti-senescence effects on human cells that are reportedly mediated by autophagy-dependent and independent pathways [144,145]. 

In addition to autophagy enhancers, the efficacy of mitophagy boosters also has been explored. For example, Nicholaos et al. [146], described a variety of pharmacological inducers of mitophagy including p62-mediated mitophagy inducer (PMI) and modulators of the PINK1/PARKIN pathway like kinetin triphosphate [147,148]. While important proof of concept has been highlighted in this review, the potential for pharmacological mitophagy activation to attenuate cardiac aging has not yet been explored.

Few pharmacological CMA activators have been identified. Humanin is a 24-amino acid mitochondrial-derived polypeptide with cytoprotective effects. The polypeptide was originally identified as a neuroprotective factor that suppressed neuronal cell death caused by Alzheimer’s disease (AD)-specific toxicity, including both amyloid-beta peptide Aβ oligomers [149]. A recent study demonstrated a cardioprotective role for humanin in the context of myocardial ischemia in mice [150]. Specifically, myocardial ischemia and reperfusion injury were attenuated by the potent humanin analogue, HNG [150]. A separate study demonstrated that humanin exerts it protective effect by activating CMA. In this regard, humanin increased the number of KFERQ-dendra fluorescent puncta per cell [151]. KFERQ-dendra fluorescent reporter changes its fluorescence pattern from diffuse to punctate when delivered to lysosomes via CMA, indicating CMA-mediated degradation and activity [152]. Further, while HNG attenuated cardiac fibrosis and reduced TGFB expression in 18 month-old female C57BL6 mice, the role of CMA was not investigated [153]. Peptide-based protein targeting technology appears to be a favorable strategy to clear the accumulation of disease-causing proteins in old hearts. For example, Fan et al. [154], reported that peptides containing the KFERQ-like motif and a protein-binding domain can be designed to specifically bind a protein of interest and direct its clearance via CMA. This approach offers an exciting possibility to engage the CMA pathway to target and degrade specific mutant and disease-causing proteins to prevent/attenuate/reverse pathology in the aging heart.

Proteasome function can be upregulated by inhibiting p38 mitogen-activated protein kinase (MAPK), its upstream regulators apoptosis signal-regulating kinase 1 (ASK1) and MAP kinase kinase 6 (MKK6), or downstream target mitogen-activated protein kinase-activated protein kinase 2 (MK2) [155]. The p38 MAPK inhibitor PD169316 was identified as the most potent activator of the proteasome by chemical genetic screening [155]. Furthermore, hormones-like epinephrine were shown to phosphorylate Rpn6 (the 19S regulatory subunit of the 26S proteasome complex) to thereby activate proteasome function in the heart and thereby increase cardiac output [156]. Thus, pharmacological strategies to activate proteasomal function appear to be beneficial in animals but translation to humans has yet to be investigated.

### 4.2. Dietary and Lifestyle Interventions

Caloric restriction (CR), defined as the reduction of calorie intake without malnutrition, is the most reproducible dietary intervention known to promote health and lifespan among model organisms [157]. CR stimulates cardiac autophagy to a greater extent in older vs. younger rodents and humans [122,158,159,160,161]. The clinical relevance of this strategy is uncertain, however, because strict compliance/self-discipline is required. Another dietary intervention approach that might be easier to implement is intermittent fasting (IF). Multiple forms of IF have been studied in the context of aging including alternate day fasting. In this regard, alternate day fasting reduced age-related cardiac hypertrophy in rats and additional indexes of cardiac aging [162]. Specifically, ad libitum feeding or alternate day fasting procedures were initiated in 2-month-old mice. Cardiac hypertrophic markers were assessed in cohorts of both groups at 6 months (young), 12 months (adult), or 24 months (old) of age. As would be predicted, sarcomeric α-actin and myosin heavy chain beta (β-MHC), and the heart failure marker B-type natriuretic peptide (BNP), were heightened in old vs. young mice that consumed chow ad libitum. As hypothesized, alternate day fasting attenuated each of these indexes of cardiac hypertrophy and heart failure [162]. In addition, evidence exists that life-long alternate day fasting enhances myocardial function and reduces oxidative stress and inflammatory markers [162,163]. One possible explanation for the benefits of alternate day fasting is via autophagy and mitophagy activation [164,165]. Similar to CR, the benefits of alternate day fasting are more pronounced when applied to old vs. young animals. Another iteration of IF is time-restricted feeding (TRF). Indeed, TRF reduces age-related cardiac dysfunction in *D. melanogaster* [166]. In addition, cardiac benefits were associated with systemic changes and tissue-specific transcriptional mechanisms [167,168]. Collectively, strong evidence indicates that simple maneuvers that require intrinsic will-power might be practical strategies for attenuating cardiac aging. The precise mechanisms responsible for these benefits (autophagy and/or mitophagy activation) have yet to be confirmed.

### 4.3. Exercise

An intervention with potential to improve autophagy and thereby influence aging-associated declines in cardiac function is Consistent dynamic exercise i.e., exercise-training. Indeed, while evidence exists that acute and chronic exercise elevate basal autophagy in normal and diseased tissues [169,170,171,172], few reports have investigated this issue in the myocardium in the context of aging. In this regard, He et al. showed that acute treadmill running increased LC3II accumulation and p62 degradation by disrupting BCL2-beclin 1 complex in the hearts of adult mice [171]. Further, Bhuiyan et al. [173] reported that voluntary wheel-running for 7 months increased cardiac autophagy and attenuated myocardial dysfunction as indicated by conserved left ventricular function in mice with desmin-related cardiomyopathy. Similarly, exercise training re-established autophagic flux and mitochondrial quality control in failing rat hearts subsequent to myocardial infarction [174]. Likewise, exercise training induced changes in autophagic function and fatty acid utilization in rabbit hearts following MI [175]. When 18 month-old mice challenged with left coronary artery ligation-induced MI were subjected to 15 min bouts of swimming, five times per week, for 8 weeks, survival rate and left ventricular function were significantly improved and myocardial fibrosis and apoptosis were reduced, when compared to age-matched mice that were sedentary [176]. While these studies clearly suggest that consistent dynamic physical exercise may improve indexes of autophagy and mitophagy in the context of aging, further in-depth mechanistic investigations are required.

## 5. Conclusions and Future Directions

This review synthesizes current literature concerning cellular proteostasis and mitochondrial clearance in the context of cardiac aging. Knowledge is evolving with regard to the contribution from and the potential for interaction among autophagy-lysosomal protein degradation pathways during the inevitable process of aging. The balance of available evidence indicates that age-associated proteotoxicity contributes to cardiac dysfunction in the mammalian heart and that this is precipitated by a defect in one or more of these highly conserved intracellular protein degradation processes together with mitophagy [5,100,115,177,178]. Despite the accumulation of information regarding cardiac proteotoxicity, important questions remain. First, at what stage in the aging process is the decline in proteolytic pathways initiated? Is the initiation and/or rate of decline similar in males and females? Because most studies in this area of inquiry have investigated models of primary aging, are defects in aging-associated protein degradation pathways and mitophagy triggered or synergized by comorbidities e.g., diabetes, hypertension or obesity? Answering these questions would shed light on when and what pathways should be targeted for possible re-activation. Second, incongruent findings exist concerning the contribution from lysosomal-mediated protein degradation in the heart. For example, while some studies show a decline in autophagy [66,84], others have indicated increased CMA activity in the hearts of aged mice [38]. These inconsistencies are likely due to the heterogeneity (e.g., age, strain, sex, methodological approaches, endpoints assessed) of preclinical models that have been used to measure lysosomal-mediated protein degradation. Third, while pharmacological and nutraceutical interventions show promise with regard to improving autophagy and mitochondrial clearance in the myocardium of preclinical models, identifying lifestyle changes with few or perhaps even beneficial off-target side effects is needed. Finally, limited data regarding the status of protein degradation pathways in aged human hearts exists. Progress on this front is challenging because of the need to complete enzymatic activity assays and organelle isolation procedures using fresh tissue, together with the requirement that both steady state indices of autophagy and indexes of autophagosome formation (e.g., autophagic “flux”) be assessed. As tools evolve, cultured human induced pluripotent stem cell-derived cardiomyocytes could be used to study autophagy, ubiquitin-proteasome protein degradation, and CMA to determine age-related alterations in protein degradation.
